# Policy approaches to improve availability and affordability of medicines in Mexico – an example of a middle income country

**DOI:** 10.1186/s12992-017-0281-1

**Published:** 2017-08-01

**Authors:** Daniela Moye-Holz, Jitse P van Dijk, Sijmen A. Reijneveld, Hans V. Hogerzeil

**Affiliations:** Department of Community and Occupational Medicine, University Medical Center Groningen, University of Groningen, Hanzeplein 1, 9713 GZ Groningen, The Netherlands

**Keywords:** Medicines, Availability, Affordability, Policy, Mexico, Middle-income country, Price negotiations, Pooled procurement

## Abstract

**Background:**

The World Health Organization recommends establishing and implementing a national pharmaceutical policy (NPP) to guarantee effective and equitable access to medicines. Mexico has implemented several policy approaches to regulate the pharmaceutical sector, but it has no formal NPP. This article describes the approach that the Mexican government has taken to improve availability and affordability of essential medicines.

**Methods:**

Descriptive policy analysis of public pharmaceutical policy proposals and health action plans on the basis of publicly available data and health progress reports, with a focus on availability and affordability of medicines.

**Results:**

The government has implemented pooled procurement, price negotiations, and an information platform in the public sector to improve affordability and availability. The government mainly reports on the savings that these strategies have generated in the public expenditure but their full impact on availability and affordability has not been assessed.

**Conclusions:**

To increase availability and affordability of medicines in the public sector, the Mexican government has resorted on isolated strategies. In addition to efficient procurement, price negotiations and price information, other policy components and pricing interventions are needed. All these strategies should be included in a comprehensive NPP.

## Background

Medicines are essential for the realization of the right to health [1]. They are one of the most effective tools to control, treat and cure diseases. Access to medicines is also one of the most sensitive indicators of the performance of a functional health system [2]. Barriers that prevent equitable and effective access to medicines limit the capacity of the system to address health issues [3]. To guarantee equitable access to medicines, the World Health Organization (WHO) has suggested the development and implementation of a national pharmaceutical policy (NPP). An NPP is “a commitment to a goal and a guide of action” for the pharmaceutical segment in the public and private sectors. Its purpose is to guarantee the availability and equitable access to medicines of assured quality, and their rational use by the population, by addressing all relevant aspects of pharmaceuticals [1]. A comprehensive NPP should address all aspects related to pharmaceuticals and medicines access, such as the legislative and regulatory framework, the selection of essential medicines as offered, the method of supply to ensure availability, rational use, affordability, the financial strategies for the optimal use of resources and the regulation of the market, the monitoring and evaluation, research and development, among others [4]. An NPP should also address potentially conflicting objectives or stakeholder interests, and should outlay the stakeholders’ roles and responsibilities [1].

Over the years, many low- and middle-income countries (LMIC) have implemented policies aimed at improving access to medicines [5]. However, in four WHO regions of the world (Africa, Eastern Mediterranean, Americas and Western Pacific), less than a third of the countries have a current and updated NPP [6]. In the Americas, 8 out of 31 countries surveyed in 2007 by WHO reported having one: Mexico, Brazil, Chile, Peru, Bolivia, Paraguay, Colombia and Venezuela [6, 7]. In many countries of Latin America (LATAM), the decentralization of health services has led to problems related to access to health care, including medicines. Even when many LATAM countries have implemented health reforms over the last years, comprehensive pharmaceutical policies have been left out [8].

Accessibility and affordability of medicines present important opportunities for improvement in many middle income countries (MIC) – including Mexico. There is some data on the availability of medicines to the population, and limited information on the affordability of these in the public and private sectors [9–12]. Studies in the late 90’s showed that about half of essential medicines were available in public healthcare facilities [13] reporting that lack of financial resources, inefficient procurement planning and distribution contributed to the unavailability of medicines [12, 13]. Other studies showed how prices of branded medicines in Mexico exceeded those in international markets [11].

Contradictory to a WHO report mentioning the availability of an NPP [7], Mexico does not have a single and concrete policy [8, 14]. In 2005 the Ministry of Health (MoH) created the background document ‘Towards an integral pharmaceutical policy for Mexico’ (Hacia una Política Farmacéutica Integral para México - HPFIM) intended to serve as a basis for an NPP but this document was never endorsed by the government [12, 15, 16]. Yet, even in the absence of an official NPP, some relevant policy instruments have been implemented addressing medicine accessibility.

Expenditure on health care constitutes 6.2% of the Gross Domestic Product (GDP) in Mexico [17]. Pharmaceutical expenditure represents 1.7% of the GDP [17, 18] and 26% [19] of the total overall health expenditure. In the public sector in Mexico, access to medicines is realized through the different social security schemes that cover formal employees, and the people’s health insurance (SPS) and the Ministry of Health (MoH) that directly provide access to medicines and health care to those without social security (mainly poor parts of the population and people in the informal sector) [20]. The federal government establishes a national formulary, and each public health provider and institution can establish its own formulary based on the national one. These institutional formularies dictate the medicines that should be accessible in the institution free of charge to the beneficiaries. Furthermore, since the health reform in 2000 that introduced the SPS in Mexico, the government has implemented policies and reforms to guarantee equitable access to health care [21]. This reform also introduced the decentralization of public procurement [8]. This allows social security and other public health institutions to procure their own medicines for their facilities, and SPS to reimburse health providers based on its catalogue of interventions and medicines that it covers. By doing so the government has tried to contain rising public spending on pharmaceuticals while guaranteeing their availability and accessibility [12] through different policies and strategies. The variation in coverage, medicines formularies and management of each public health institution and the introduction of the SPS has led to a fragmented system with differences in access to medicines and in the efficient use of resources among institutions [20].

The present study aims to describe the approach that the Mexican government has taken to improve accessibility in the last decade. A descriptive policy analysis was conducted by identifying and describing the strategies and actions proposed and implemented by the government aimed at improving availability and affordability of pharmaceuticals.

## Methods

**Fig. 1:**
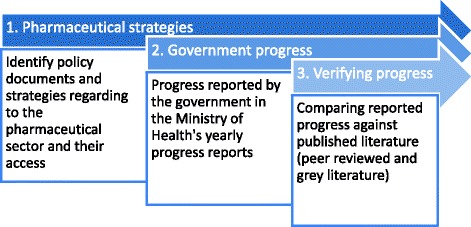
Flow chart of the documents reviewed for the analysis of policies regarding medicines

This descriptive policy analysis follows an inductive research approach, with government reports and grey and peer-reviewed literature as data sources. The study was conducted in three stages, as shown in Fig. 1.

First, relevant government pharmaceutical policies and strategies from 2005 to 2013 were identified and compared. Objectives, strategies and proposed lines of action on accessibility and affordability were identified. Three focus areas were selected: procurement, pricing, and information on medicines. Second, MoH’s yearly progress reports from 2007 until 2015 were studied, to collect data on affordability and accessibility of medicines. These data were compared with the policies and strategies previously identified. Thirdly, a literature review was conducted with the following keywords: Mexico, medicines, access to medicines, access to treatments, medicines policies, pharmaceutical policies, availability and affordability of medicines, medicines prices, price negotiations, Coordinating Commission for the Negotiation of Prices of Medicines (Comisión Coordinadora para la Negociación de Precios de Medicamentos - CCNPMIS), procurement of medicines, national formulary, essential medicines list, basic scheme and catalogue for medicines and health devices (CBMCIS), pooled procurement, supply of medicines. The literature search was conducted in Google and Google Scholar, the websites of the MoH, Pan American Health Organization (WHO/PAHO), National Institute of Public Health in Mexico (INSP), BVS (virtual library of Mexico in Health), OECD, Fundación para la Salud (FUNSALUD), PubMed, and grey literature from various sources.

The following inclusion criteria were used: publication date between 2005 and 2016; the document referred to Mexico; the document presented data and/or opinions focusing on medicines affordability, accessibility and/or availability of medicines, and medicines policies. Approximately 40 documents were identified, of which 26 were used.

Data analysis included verifying whether progress on the strategies reported by the government responded to the strategies and lines of action proposed. Grey literature served to support this comparison and to provide further input.

## Results

### Key pharmaceutical policy documents and the development of an NPP

In 2005 the HPFIM document was issued to serve as a basis for a national pharmaceutical policy. The next government (2007-2012) adopted some aspects of this document related to medicines access in its health action plan (PROSESA) for 2007-2012 [14]. In its action 3.6, the government committed to developing an NPP that promotes the efficient and timely supply of medicines. In 2007 the representatives of the National Executive and Legislative Powers, together with relevant stakeholders, signed the “Commitment to guarantee the sufficiency, availability and fair prices of medicines” [22]. The commitment outlined 14 lines of action. Some were used in successive governmental health plans [23] but no formal and comprehensive policy was published and implemented.

**Fig. 2:**
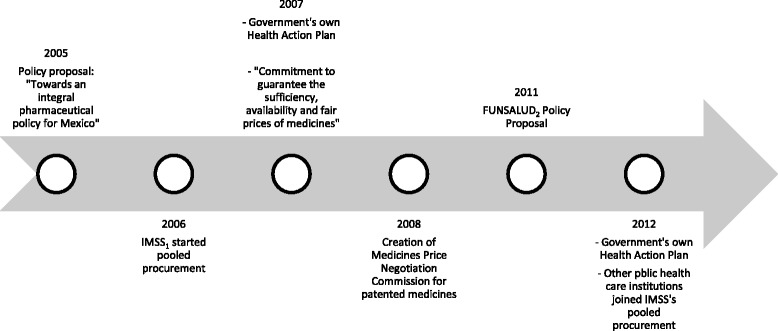
Timeline of proposals and actions to establish a National Pharmaceutical Policy and strategies to improve affordability and availability of medicines: 2005 to 2015

In 2011, the civil association FUNSALUD analyzed the pharmaceutical sector in Mexico, and provided policy proposals with more specific strategies and lines of action [24]. This document also remained a proposal. The current government’s health action plan for 2013-2018 [25] set its own strategies and lines of action. Figure 2 presents a timeline with the policy documents, strategies and proposals developed during the time frame of 2005-2015 that aimed to develop an NPP.

The policy documents issued in 2005 and 2011 remained as proposals and no formal and single NPP, addressing all pharmaceutical access aspects in a comprehensive manner, was developed. However, we identified isolated policies and strategies addressing several aspects of medicines. We further describe the policies issued in the last years that have aimed to improve availability and affordability.

### Availability and procurement of medicines

HPFIM and both health plans acknowledge the need for strategies to improve medicine availability in the public sector. One of the main strategies promoted is pooled or consolidated procurement of multi-source medicines, with the goal of maintaining efficiency of public procurement and also monitoring the level of compliance with generic policies.

In 2006, the Mexican Institute of Social Security (Instituto Mexicano del Seguro Social - IMSS) started consolidating the procurement of multi-source medicines, setting maximum reference prices [26, 27] for public procurement. IMSS’s main procurement objective has been low prices [27, 28]. This approach has brought considerable savings but some observers state that it “has been prone to low quality and non-compliance of the providers” [26, 28]. In 2011, the OECD reported that centralized medicines procurement had a 30% contract non-compliance rate on quality and delivery of goods from providers in some isolated regions in the country [26]. Since 2012, other social security health institutions and public hospitals have joined the consolidated procurement led by IMSS [29]; some health ministries of states and federal hospitals are also joining gradually. During the annual procurement rounds of 2013-2015 the government reported accumulated savings of $10,863 million Mexican pesos (MXP) (US$776.78 million) [30–32].

The government defines availability as “the percentage of patients that got their medicine prescriptions completely filled in the public sector” and reported a slight increase from 78% in 2003 to 80.1% in 2010 [33]. However, the probability of obtaining medicines still varies depending on the health institution and states [34–36]. According to the 2012 National Health and Nutrition Survey [37] 92% of out-patient users received their prescribed medicines. Of these patients 65.2% received their medicines at the pharmacy of their health care unit the day of their doctor’s appointment, while the rest had to go to another facility or come back another day(s) to fill their prescriptions. The survey reported that 66-86.1% of the patients using social security services got their prescriptions completely filled. As for state health services (Servicios Estatales de Salud -SESA), 63.7% of patients received their prescribed medicines [37]. Patients with social security are more likely to get their prescriptions filled than those in SESAs, which are affiliates of the People’s Health Insurance (Seguro Popular de Salud, SPS) and patients without any health insurance (non-SPS users) [34, 38, 39]. These data were reported before 2012 when the pooled procurement was extended. Since then no data have been published on actual availability of medicines and on the impact of the pooled procurement strategy on access to medicines in the public sector.

### Prices of medicines

According to HPFIM, medicine prices in Mexico are one of the highest in LATAM countries [10, 40–43]. Chaumont et al. reported that for several originator antiretrovirals, like lopinavir + ritonavir, atazanavir, and tenofovir, prices in Mexico are higher than other middle-income countries and sometimes even higher than high-income countries [44]. In a survey of affordability and availability of medicines in Mexico City the prices of several generic and originator brand products were 2 to 5 times higher than in other LATAM countries [10]. According to Danzon and Furukawa [45, 46] who compared medicine prices among several countries, “when prices are adjusted according to income, Mexicans are paying higher prices than people in other countries” [10, 15, 46, 47].

Since 2004, following the agreement between the Mexican government and the pharmaceutical industry, the mechanism used to contain medicine prices has been limited to patented medicines. The prices of multi-source medicines are no longer regulated and follow free market competition [15]. In the current pricing scheme, each pharmaceutical company sets the maximum retail price (MRP) of its products. Participation to the scheme is voluntary [10, 15, 40, 47]. If companies fail to adhere to the scheme, they could be subject to other price control mechanisms when a lack of effective competition exists [15]. To define the MRP, a company takes the average price of at least 6 countries, weighed from the actual sales volume, for the last 3 months of the date of the proposed MRP registration [15]. The price in Mexico, together with the price-setting report and how the company will calculate future price increases, is reported to the Ministry of Economics (MoE) and must be printed on the package [15].

In 2015 the Senate submitted a new initiative regarding medicine prices [40]. This project included a reform to the General Health Law – an amendment of articles 17^bis^ and 31, and the inclusion of articles 31 ^bis^ and 233 ^bis^. The reform to Article 31 proposed that the MoE should fix the MRPs of medicines, after hearing the MoH. Article 31^bis^ proposed to set the MRP on the basis of external reference pricing (ERP). Article 17^bis^ introduced surveillance on the compliance of articles 31 and 31^bis^. Article 233^bis^ introduced an information system for medicines prices and availability in pharmacies, to be included on the national regulatory agency website. However, these proposed reforms were not accepted in the law [48].

In 2008 a coordinating commission for price negotiations (CCNPMIS) was created to end the variance in prices paid for the same product in the public sector, to reduce expenditure on single-source medicines, and to increase their accessibility. It negotiates public procurement prices of single-source products included in the national formulary [49]. The negotiations take place on a yearly basis. The negotiating criteria include the therapeutic benefits with respect to other alternatives, and the estimated consumption volume [50].

**Fig. 3:**
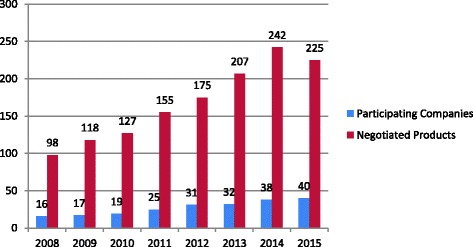
Number of companies participating in the Coordinating Commission for the Negotiation of Prices of Medicines Negotiation process and number of products negotiated

From 2008 to 2015 the number of participating companies and the number of negotiated products has increased, as reported in the annual government reports (Fig. 3). The CCNPMIS has contributed to standardizing the prices for patented medicines in the public health sector [51]. Within its first 5 years of operation, it obtained important savings and has prevented price increases [42, 52]. Over the seven negotiating rounds, the authorities estimate savings close to 18,000 million MXP (US$1417million) [30] on the expenditure of medicines [53].

Any price reduction achieved by the CCNPMIS can be considered as a success [54]. Yet the commission also faces significant challenges, such as poor coordination and lack of communication among the institutions with respect to the timely preparation of background materials required for the negotiations. There is a shortage of staff members with the necessary technical expertise [29, 52], poor management of the annual negotiation process, lack of political support, and lack of explicit indicators for assessing its performance [52].

### Improving availability and prices of medicines through information systems

In 2011 the government implemented the Sectorial Centre of Web Management of Medicines Information (CesMed) to further improve procurement practices and prevent large variations in prices. CesMed aims to provide states and public health institutions information about medicines included in the national formulary, comparative prices and volumes, and the best procurement practices. By 2015 CesMed had captured information from approximately 70 institutions. However it is not mandatory for institutions to report and share information. This has limited the collection of timely and complete information. The system does not operate in real time, further limiting its use for decision making. Information management continues to be segmented and each institution manages their information independently [55, 56].

## Discussion

We analyzed the strategies the Mexican government has used to improve availability and affordability of medicines. Since 2005 these efforts have focused on pooled procurement of multi-source medicines, central price negotiations of patented medicines, and the creation of an electronic information platform in the public sector. The government has limited the reporting on the impact of these initiatives to overall savings on expenditure without much supporting evidence.

We started by reviewing policy documents and proposals that aimed at the development of an NPP. Several authors [8, 57–59] have analyzed the development of pharmaceutical policies by various countries, reporting on the complexity of such process and how this development is specific according to each country’s settings and circumstances. Hobert et al. reported that LMIC are more likely to develop an NPP, as a comprehensive policy is more inclined to address pharmaceutical problems and improve access to medicines, than single policy components [57] as in the Mexican case. For many LMIC pharmaceutical policies have come as a part of an overall health reform [58]. These policies have mostly aimed at a more efficient and rational use of resources as well as reaching equity [8, 58, 59], while accommodating stakeholders concerns and roles on access to medicines [8]. Thus, the WHO has promoted the implementation of an NPP and has provided support to LMIC to develop their own [1]. Up to date no single and comprehensive NPP has been developed in Mexico. Instead, it has been developed in isolated components, found in several laws, policies and procedures [57].

### Availability of medicines

We found that pooled procurement has generated estimated savings of about 10% of the pharmaceutical expenditure. However, improvement in availability of medicines has not been assessed. Experiences with PAHO’s Revolving Fund [60, 61], and centralized procurement mechanisms used in Brazil [62], Argentina, Uruguay, and in Chile [63, 64] have shown that by consolidating product requirements and increasing purchase volumes, purchasers can benefit from economies of scale leading to lower prices and increasing procurement efficiency [61, 65]. Thus, the extended use of pooled procurement has indeed the potential to promote efficiency and reduce variations in medicines prices within the Mexican public sector. In order to assure the availability of medicines in public health care facilities in Mexico, a more comprehensive assessment of the procurement process is needed, considering the product specifications, contractual conditions of delivery, and sanctions in case of non-compliance. Suppliers invited to tender should first be pre-qualified [66] to ensure the quality of their goods and the reliability of the delivery process. It is also important to monitor the impact of the pooled procurement on the ultimate availability of medicines, in comparison to previous years, and to monitor supplier performance.

### Affordability of medicines

We found that for single source medicines in the national formulary, the government has introduced price negotiations [56]. This follows examples in other countries, such as Canada and Western Europe, where monopsony purchase and bargaining powers have kept prices low [66]. Hospitals in Colombia carry out joint negotiations [67] and Brazil has included in its law provisions on patents and intellectual property that have helped to improve its negotiating power [68–70]. In Mexico, progress reports on the impact of the negotiations have focused on discounts and savings achieved, which can be estimated at around 7% of pharmaceutical expenditure. However, it is not clear whether the negotiated final prices have been respected during procurement procedures [71] and whether access to these medicines has actually improved. Some challenges need to be addressed to improve the role of the CCNPMIS. Its performance needs to be assessed thoroughly using selected indicators [52]. Political support is required to institutionalize the commission – as already proposed by FUNSALUD [24] – and to strengthen its procedures.

### Pricing regulation

In Mexico, a pricing scheme for medicines in the private sector is in place. However, there is no evidence that it is adequate [9, 15, 47] since the government does not establish price controls itself. Instead, the industry establishes the maximum retail price itself. This scheme may follow a free market philosophy, but it is not in line with WHO recommendations on price controls [72, 73] where a much more active role of the government is recommended. Several LATAM countries, such as Brazil and Columbia, have implemented price regulation policies based on external reference pricing mechanisms [62, 67, 74, 75]. Mexico should also consider moving towards active price regulation [47], to improve the affordability and accessibility of medicines.

Price regulation and price negotiations as individual measures are not likely to be successful. These should be part of a comprehensive medicine policy. For example, the country could implement internal and external reference pricing for multi-source (generic and branded generic) and single-source (often patented originator brand) medicines. It should also encourage the use of voluntary licenses to increase competition, and issue compulsory licenses in case the industry is not collaborating. Other measures can also contribute to better affordability, such as those related to cost-effective prescribing and therapeutic (as opposed to generic) substitution. A full range of possible strategies to reduce prices has been described elsewhere [73, 76–79]. Most of these measures could be incorporated into the Mexican legislation to improve affordability of medicines.

### Procurement and pricing information

We found that participating institutions and states have shared valuable information and success stories in the CesMed platform that can be helpful for other institutions facing procurement or pricing problems. Such platform can indeed facilitate the implementation, monitoring and assessment of procurement and prices of medicines and of a NPP [80]. However, its potential is currently limited as only few institutions use it. Brazil, Colombia, Argentina and Peru have also implemented medicines’ pricing information systems to monitor medicines prices, with the ultimate goal to increase transparency and improve access [64, 74, 81, 82]. These systems could serve as examples for Mexico.

In the 2011 policy proposal, FUNSALUD proposed to monitor public prices of medicines to increase the efficiency of public procurement and the use of resources [24]. CesMed could provide such platform, but all public health institutions should participate in a timely manner and with a uniform report format. Increasing access to this information should allow purchasers to compare their own prices, obtain better prices, and better estimate their budget requirements [64]. More transparency on pricing also helps to reduce and prevent corruption.

### Strengths and limitations

This study is based on reported information provided by the government, providing a scope of the actions that Mexico has taken towards improving access to medicines. The main strength of this study is an extensive search for policy and governmental documents related to availability and affordability of medicines, in addition to the analysis of the governmental yearly health reports. However, an official document cannot provide judgement on availability and affordability. No detailed data on medicines affordability and availability are publicly available to fully assess the government’s strategies. In our research we did not try to study actual availability and affordability on the basis of policy documents, but to identify and describe strategies and policies aimed to improve availability and affordability. Our data cannot provide a comprehensive analysis of the policies’ outcomes and shortcomings. Moreover, the information reported by the government may have been too positive or optimistic on the effect of these strategies on access to medicines and generated savings, without fully disclosing their real impact on actual availability and affordability. Thus our findings may underestimate somewhat the current availability and affordability.

We think that explanations for the limited success of these strategies include the lack of monitoring during implementation and lack of adaptation of the strategies over time to achieve the desired outcomes. Moreover, lack of transparency, some conflicts of interests of stakeholders and corruption may have affected the actual outcomes of these strategies and the development of a full NPP.

### Practical implications

We found that in 2007 the government committed itself to establishing an official NPP [22, 23]. Some aspects mentioned in the policy proposals of 2005 [43] and 2011 [24] have indeed been included in health action plans and have been followed up. Yet, an official and comprehensive NPP [12, 14, 16] addressing all medicine-related aspects of a health system [1, 57] is still lacking. In view of the many different stakeholders and the need for a comprehensive approach to further improve medicine availability and affordability, the official development, adoption and implementation of an NPP is very much needed. Experiences in other countries have shown that an NPP, developed in a consultative manner with all involved stakeholders, should provide a common ground of action with the main goal of guaranteeing access to medicines [57, 83].

The government has implemented isolated policy components to improve availability and affordability of medicines. Although desired outcomes have been achieved, all these approaches have shown limitations in their implementation. Most public health institutions have joined to increase the procurement volume and achieve savings through pooled procurement, yet actual unavailability has been reported due to providers’ non-compliance. This issue is related to lack of monitoring. Also for price negotiations, the government has not monitored the actual procurement prices of the negotiated medicines, to truly assess the impact of this strategy.

Further independent research is necessary to evaluate the effect of government strategies, to identify their strengths and weaknesses, and measure their final impact on access to essential medicines. We recommend a comprehensive examination of the procurement process; the institutionalization of the negotiating commission; and increase information reporting through the CesMed platform.

## Conclusions

To increase the availability and affordability of medicines in the public sector, Mexico has focused on pooled procurement of multi-source products, price negotiations for single-source products and collection and dissemination of price and procurement information. The government claims that these strategies have provided considerable savings. The real impact of these strategies on actual availability and affordability has not been independently assessed. These three strategies have been developed and implemented in isolation, limiting their potential impact. Ample room exists to improve the availability and affordability of drugs in Mexico. All strategies should be included in one comprehensive NPP aimed not only at improving medicines accessibility and affordability, but also at other aspects, such as medicines quality, safety and quality use.

## Abbreviations

BVS: Biblioteca Virtual en Salud - Virtual library of Mexico in health
CBMCIS: Cuadro Básico y Catálogo de Medicamentos - Scheme and catalogue for medicines
CCNPMIS: Comisión Coordinadora para la Negociación de Precios de Medicamentos - Coordinating Commission for the Negotiation of Prices of Medicines
CesMed: Centro Sectorial de Gestión Web de Información de Medicamentos - Sectorial Centre of Web Management of Medicines Information
ERP: External Reference Pricing
FUNSALUD: Fundación para la Salud - Foundation for Health
GDP: Gross Domestic Product
HPFIM: Hacia una Política Farmacéutica Integral para México - Towards an integral pharmaceutical policy for Mexico
IMSS: Instituto Mexicano del Seguro Social - Mexican Institute of Social Security
INSP: Instituto Nacional de Salud Pública - National Institute of Public Health
LATAM: Latin America
LMIC: Low- and Middle-income countries
MIC: Middle-income countries
MoE: Ministry of Economics
MoH: Ministry of Health
MRP: Maximum Retail Price
MXP: Mexican Pesos
NPP: National Pharmaceutical Policy
OECD: Organization for Economic Co-operation and Development
PAHO: Pan American Health Organization
PROSESA: Programa Sectorial de Salud - Health Action Plan
SESA: Servicios Estatales de Salud - State health services
SPS: Seguro Popular de Salud - Poeple’s Health Insurance
WHO: World Health Organization
